# Family Caregiving in the Somali-American Community During COVID-19

**DOI:** 10.1093/geroni/igab046.3395

**Published:** 2021-12-17

**Authors:** Rajean Moone, Kamal Suleiman, Elizabeth Lightfoot

**Affiliations:** 1 University of Minnesota, Woodbury, Minnesota, United States; 2 University of Pennsylvania, Woodbury, Minnesota, United States; 3 Arizona State University, Woodbury, Minnesota, United States

## Abstract

This poster describes a study of Somali American family caregiving during the COVID-19 pandemic, specifically investing the unique caregiving challenges faced by Somali caregivers. The findings from this study, which was part of a larger study related to family caregiving, were drawn from in-depth interviews of ten Somali family caregivers in Minnesota. All interviews were conducted in Somali during the summer of 2020 and translated and transcribed by a certified translator and research assistant. The major themes that emerged from this study related to Visitation, Hospital Accompaniment, and Self Sacrifice. As Somali culture is centered around extended family connections, isolation places particular strain. Second, family members typically serve as advocates, translators and guides during hospital visits. The restrictions on hospital accompaniment due to COVID-19 caused increased stress and poorer care of family caregivers and their loved ones. Third, in the Somali community, family placement is a last resort. Caregivers reported great sacrifices in time, comfort and opportunities to care for family members during COVID-19. Most of the caregivers described their coping with these sacrifices in terms of radical acceptance and God consciousness. These findings have important implications for providing supports for Somali family caregivers.

